# Brain core temperature of patients with mild traumatic brain injury as assessed by DWI-thermometry

**DOI:** 10.1007/s00234-014-1384-5

**Published:** 2014-07-12

**Authors:** Jun Tazoe, Kei Yamada, Koji Sakai, Kentaro Akazawa, Katsuyoshi Mineura

**Affiliations:** 1 Department of Radiology, Graduate School of Medical Science, Kyoto Prefectural University of Medicine, Kajii-cho, Kawaramachi Hirokoji Agaru, Kamigyo-ku, Kyoto City, Kyoto 602-8566 Japan; 2 Department of Human Health Science, Graduate School of Medicine, Kyoto University, Kyoto, Japan; 3 Department of Neurosurgery, Graduate School of Medical Science, Kyoto Prefectural University of Medicine, Kyoto City, Kyoto Japan

**Keywords:** MRI, Brain, Diffusion-weighted imaging, Lateral ventricular temperature, Mild traumatic brain injury

## Abstract

**Introduction:**

The aim of this study was to assess the brain core temperature of patients with mild traumatic brain injury (mTBI) using a noninvasive temperature measurement technique based on the diffusion coefficient of the cerebrospinal fluid.

**Methods:**

This retrospective study used the data collected from April 2008 to June 2011. The patient group comprised 20 patients with a Glasgow Coma Scale score of 14 or 15 who underwent magnetic resonance imaging within 30 days after head trauma. The normal control group comprised 14 subjects who volunteered for a brain checkup (known in Japan as “brain dock”). We compared lateral ventricular (LV) temperature between patient and control groups. Follow-up studies were performed for four patients.

**Results:**

LV temperature measurements were successfully performed for both patients and controls. Mean (±standard deviation) measured LV temperature was 36.9 ± 1.5 °C in patients, 38.7 ± 1.8 °C in follow-ups, and 37.9 ± 1.2 °C in controls, showing a significant difference between patients and controls (*P* = 0.017). However, no significant difference was evident between patients and follow-ups (*P* = 0.595) or between follow-ups and controls (*P* = 0.465).

**Conclusions:**

A reduction in brain core temperature was observed in patients with mTBI, possibly due to a global decrease in metabolism.

## Introduction

Brain temperature is determined by the balance between heat generation and heat removal, involving three essential elements: brain metabolism, cerebral blood flow, and body core temperature [1]. Heat generation results from neuronal activity, and this heat is mainly removed from the cranium by the abundant blood flow to the brain. Conversely, blood has its own temperature similar to that of the body core, so brain temperature is usually approximately 1 °C higher than body core temperature. Physical activity or pyrexia will result in an increase in body core temperature, which will subsequently increase brain temperature.

Severe head trauma causes direct destruction of the brain parenchyma, metabolic dysfunction, and a decrease in blood flow [1]. In patients with severe head trauma, a Glasgow Coma Scale (GCS) score of <8 is known to be associated with increased brain temperature, because cerebral blood flow is decreased, limiting the heat removal function [2]. The brain temperature of patients in mild traumatic brain injury (mTBI), on the other hand, has not been extensively studied.

Various methods are available to evaluate brain temperature. The most precise is the measurement of intracranial temperature during cranial surgery for hematoma removal or intracranial pressure reduction [3, 4]. This method, however, is invasive and unsuitable for application to patients with mTBI. Tympanic and/or rectal temperature measurements have been substituted as indicators of brain temperature [3, 4], but the accuracy of these methods has yet to be established [5, 6]. Several noninvasive techniques are now available, including microwave radiometry [7] and magnetic resonance (MR) imaging [8–11]. For instance, methods of brain temperature measurement based on T1-weighted imaging [8] or MR spectroscopy (MRS) [9–11] have been established. These methods, however, cannot be performed routinely in the clinical setting, due to the time required for imaging and postprocessing. Recently, a novel method using diffusion-weighted imaging (DWI) has been proposed [12]. This method calculates the temperature of the cerebrospinal fluid (CSF) based on the measured diffusion coefficient of the lateral ventricles [13–16].

Recent clinical studies have identified that brain metabolism decreases temporarily in patients after mTBI [17–20]. We postulated that this decrease in metabolism might lead to the decline in brain temperature. If we could correlate a decrease in brain metabolism to reductions in lateral ventricular (LV) temperature using this DWI method, we might be able to evaluate the brain condition of patients with mTBI in greater detail. This method might thus facilitate the clinical management of patients with mTBI. The purpose of this study was thus to retrospectively assess the cerebral temperature of patients with mTBI.

## Methods

### Patient and control populations

A retrospective search of the radiology reporting system in our institution from April 2008 through June 2011 revealed 23 patients who had undergone MR examination after head trauma. Each MR examination was performed within 20 days (days 0–19; mean (±standard deviation (SD)), 4.3 ± 5.2) of injury. Within this patient population, 20 met the inclusion criteria for the definition of mTBI, an initial GCS score after 30 min of 13–15, loss of consciousness (LOC) for approximately ≤30 min, posttraumatic amnesia (PTA) for ≤24 h [17], and lack of LV hemorrhage on T2*-weighted imaging. Patients with gross ventricular hemorrhage were excluded, as the increased protein content and resultant change in CSF viscosity could lead to errors in estimating temperature. Mean patient age was 63.0 ± 15.8 years (range, 21–85 years). Among the 20 patients, follow-up MR images were available for four patients, with all follow-up examinations performed >30 days after injury (mean, 126.0 ± 64.3 days; range, 43–200 days).

The control group comprised 14 healthy, age-matched adults (mean age, 56.4 ± 15.3 years; range, 28–75 years; 3 men, 11 women) who voluntarily participated in a brain checkup. This is also known as “brain dock” in Japan, where some healthy adults choose to undergo brain examinations at their own expense because they are concerned about certain diseases, such as cerebral aneurysm. All MR images were reviewed by board-certified radiologists and diagnosed as normal. This study was approved by the review committee of our institution, and written informed consent for MR examinations was obtained from all subjects prior to participation in this study.

### Data acquisition

All MR examinations were performed using a 1.5-T whole-body scanner (Philips Medical Systems, Best, the Netherlands). DWI was performed with an image acquisition time of approximately 3 min. A single-shot echo-planar imaging technique was used for DWI with the following parameters: repetition time (TR), 6,000 ms; echo time (TE), 88 ms; *b* value, 1,000 s/mm^2^; image averaging, two times; field of view (FOV), 230 mm; motion-sensitizing gradients applied in 15 directions; and 42 3-mm-thick sections obtained without intersection gaps.

The other conventional MR images obtained in this study included T2*-weighted images (gradient echo sequence; TR, 755 ms; TE, 23 ms; 5-mm-thick sections with 1-mm gap; FOV, 230 mm) and fluid-attenuated inversion recovery (FLAIR) images (delay time, 2,200 ms; TR, 8,000 ms; TE, 100 ms; 5-mm-thick sections with 1-mm gap; FOV, 230 mm).

### Temperature estimation

The kinetic theory states that a direct relationship exists between the absolute temperature and the diffusion coefficient. The diffusion coefficient of nonrestricted water molecules can be reliably measured using MR [18]. Recent studies by Mills [19] and Kozak et al. [12] showed that the temperature of the CSF can be estimated using this relationship. Mills [19] reported the relationship between pure water diffusion and diffusivity. Kozak et al. [12] demonstrated the ability of DWI to measure the temperature of the CSF based on the work of Mills [19]. Based on those studies, we determined LV temperature by calculating the diffusion constant using Eq. 1:

1 $$ D=\frac{ \ln \left({S}_0/ S\right)}{b} $$

where *D* is the diffusion constant (square millimeters per second), *b* is the applied diffusion weighting (seconds per square millimeter), and *S*_0_ and *S* are the voxel signal intensities of the reference and diffusion-weighted images, respectively. The *D* value was converted to the corresponding temperature by Eq. 2:

2 $$ T=\frac{2256.74}{ \ln \left(\frac{4.39221}{D}\right)}-273.15 $$

where *T* is the temperature (degrees Celsius). This temperature estimation is only considered to apply within the lateral ventricles, since this method is only applicable for nonrestricted water. This DWI-based MR thermometry of the ventricles was performed using custom-developed software.

### Body temperature

Body temperatures of patient and control groups were compared, as brain temperature could be affected by the body temperature. Axillary temperature (C202 axillary thermometer; Terumo, Tokyo, Japan) was used for the patient group, measured as part of the bedside routine on the same day as MR imaging. For the control group, we used tympanic temperature (EM-30CPLB infrared ear thermometer, Terumo), which had been measured in the room next to the MR scanner. The difference in these measurement methods is due to the fact that this was a retrospective study only planned after the completion of volunteer scans. Because axillary temperature has been reported to be approximately 0.2 °C lower than tympanic temperature [20], upon comparison, we added 0.2 °C to each axillary temperature measurement from the patient group. No upgrade of the MR scanner was performed during the study period nor was there any change in the thermometers used.

### Statistics

Statistical analysis was performed using a nonparametric Wilcoxon rank-sum test (SPSS version 19; SPSS, Chicago, IL, USA) to compare the patient and control groups regarding LV and body temperatures and follow-up and control groups regarding LV temperature. To compare the patient and follow-up groups regarding LV temperature, the Wilcoxon signed-rank test was used. Correlations with values of *P* < 0.05 were evaluated as significant.

## Results

Twenty patients (15 men, 5 women) met the inclusion criteria for this study. Causes of head trauma were falls in 18 patients (90 %; a fall under unknown circumstances in 15 cases (75 %), from bed in 1 (5 %), from a height of about 1 m in 1 (5 %), and down a stairway in 1 (5 %)). In the remaining two patients, one was hit in the face by a softball (5 %) and the other was involved in a motor vehicle accident (5 %). Six of the 20 patients were under the influence of alcohol on arrival at hospital. When MR imaging was performed, all patients were sober because ≥1 day had passed since admission. All patients showed GCS 15 at the time of MR examination.

The majority of patients (90 %) showed positive MR findings, with subarachnoid hemorrhage in 9 (45 %), subdural hemorrhage in 8 (40 %), brain contusion in 7 (35 %), extradural hemorrhage in 5 (25 %), skull fracture in 2 (10 %), pneumocephalus in 2 (10 %), and blowout fracture in 2 (10 %). Patient data are summarized in Table 1.

**Table 1:** Patients in mild traumatic brain injury

No.	Age	Sex	GCS*1	MRI				Cause of injury	Findings
				Days	GCS*2	AX TEMP [°C]	LV TEMP [°C]		
1	69	M	15	1	15	37.0	37.5	Fall	SAH, SDH
2	78	F	15	0	15	36.7	36.4	Fall	BC, SDH, BOF
3	49	M	14	1	15	36.2	36.0	MVA	None
4	62	M	15	1	15	37.0	36.5	Fall	SF
4f			–	43	15	–	37.9		
5	63	M	15	13	15	36.6	36.3	Fall	SF, SDH
5f			–	130	15	–	39.3		
6	55	M	15	19	15	36.6	38.6	Fall	SF, BC, EDH
6f			–	131	15	–	36.7		
7	52	M	14	1	15	36.6	35.4	Fall	SF, BC, EDH
8	60	M	14	2	15	38.0	36.2	Fall	SF, BC, SAH, EDH, PC
9	39	M	15	3	15	37.0	35.0	Fall	SF, SDH
10	75	M	14	6	15	36.4	36.1	Fall	SAH, SDH
11	69	M	14	1	15	37.0	36.9	Fall	BC, SAH
12	82	F	15	9	15	36.4	36.6	Fall	EDH
13	85	F	15	0	15	36.8	36.4	Fall	SAH
14	72	M	14	5	15	36.4	39.1	Fall	EDH
15	80	F	15	11	15	36.5	40.1	Fall	BC, SAH, SDH
15f			–	200	15	–	40.8		
16	66	M	14	1	15	36.7	36.9	Fall	None
17	46	M	15	1	15	36.2	36.6	Hit by a softball	BOF
18	21	F	15	1	15	36.8	35.8	Fall	SAH, SDH
19	64	M	15	8	15	37.2	40.2	Fall	SF, SAH, PC
20	72	M	15	2	15	36.6	35.7	Fall	BC, SAH, SDH

*GCS* Glasgow Coma Scale, GCS*1 primary GCS, GCS*2 GCS when MRI was performed, *Days* days after head trauma, *AX TEMP* axillary temperature, *LV TEMP* lateral ventricular temperature, *4f, 5f, 6f, 15f* follow-up cases, *M* male, *F* female, *MVA* motor vehicle accident, *SDH* subdural hemorrhage, *BC* brain contusion, *BOF* blowout fracture, *SF* skull fracture, *EDH* extradural hemorrhage, *PC* pneumocephalus

**Fig. 1:**
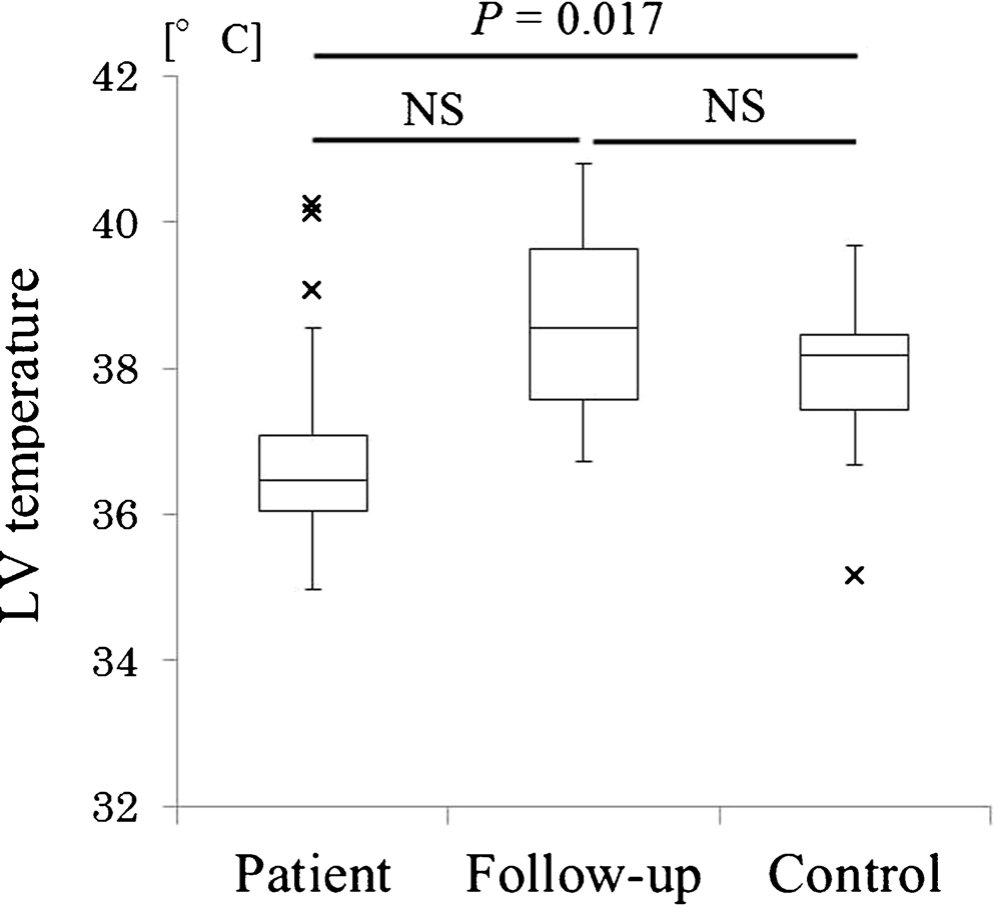
LV temperature in each group. A significant difference was apparent between the patient group undergoing MRI after head trauma and the control group (*P* = 0.017). Conversely, no significant difference was seen between patient and follow-up groups (*P* = 0.465) or between control and follow-up groups (*P* = 0.595). The *multiplication sign* indicates outlier. *NS* no significant difference

Mean (±SD) LV temperature was 36.9 ± 1.5 °C in the patient group (range, 35.0–40.2 °C; median, 36.5 °C), 38.7 ± 1.8 °C in the follow-up group (range, 36.7–40.8 °C; median, 38.6 °C), and 38.0 ± 1.2 °C in the control group (range, 35.2–39.7 °C; median, 38.2 °C) (Table 2). These results are presented in Fig. 1. A significant difference was evident between the patient and control groups, with LV temperature approximately 1.1 °C lower in the patient group (*P* = 0.017). On the other hand, no significant difference was seen between the patient and follow-up groups (*P* = 0.465) or between the control and follow-up groups (*P* = 0.595).

**Table 2:** Normal control group

No.	Age	Sex	Tympanic TEMP [°C]	LV TEMP [°C]
1	28	F	36.6	39.7
2	32	F	36.9	38.4
3	41	M	36.5	36.7
4	43	M	36.6	36.9
5	49	M	36.7	37.7
6	55	F	36.6	38.7
7	61	F	36.2	38.5
8	64	F	36.3	35.2
9	65	F	36.9	38.1
10	67	F	36.6	39.5
11	67	F	36.7	37.3
12	70	F	36.8	38.4
13	73	F	35.9	38.3
14	75	F	36.7	37.9

*Tympanic TEMP* tympanic temperature, *LV TEMP* lateral ventricular temperature, *M* male, *F* female

**Fig. 2:**
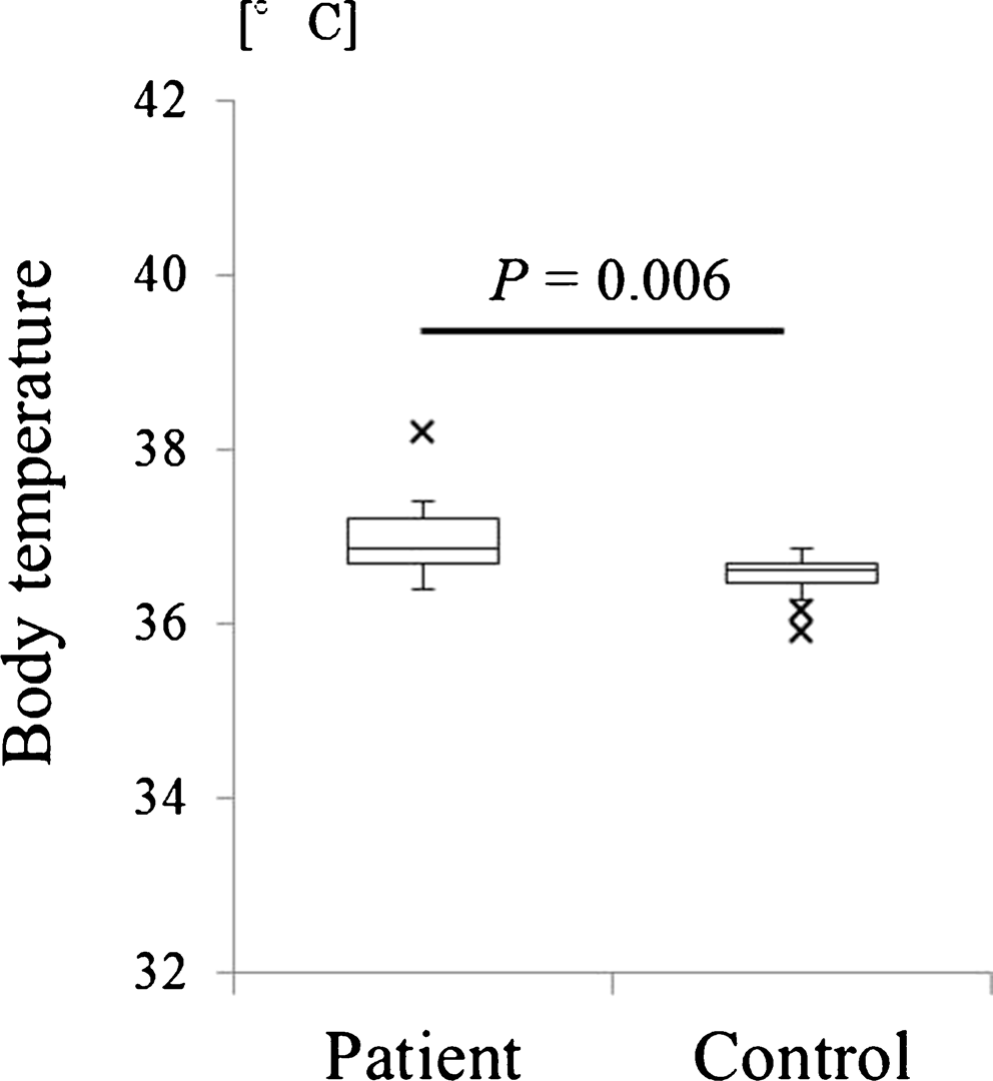
Body temperature in the patient and control groups. A significant difference was seen between groups (*P* = 0.006), with body temperature approximately 0.3 °C higher in the patient group. The *multiplication sign* indicates outlier

Mean axillary temperature of patients after head trauma was 36.7 ± 0.4 °C. After adding 0.2 °C for adjustment, the adjusted values were 36.9 ± 0.4 °C for patients and 36.6 ± 0.3 °C for controls (Fig. 2). A significant difference was also evident between the patient and control groups (*P* = 0.006), with body temperature being approximately 0.3 °C higher in patients following head trauma.

## Discussion

Most of what we know about TBI comes from the literature regarding cases of moderate-to-severe TBI and not from mild trauma cases. In cases of severe head trauma, brain temperature is known to generally increase [2]. The temperature elevation has been attributed to higher intracranial pressure leading to higher resistance, consequently reducing cerebral blood flow. Very little is known about the pathophysiology of mTBI, and this study showed a trend toward decreased brain temperature in such cases. This is conceivably due to changes in global metabolism, perfusion, or both combined. Global declines in metabolism have been reported using various methods, including MRS. A few studies have shown a metabolic decline in patients with mTBI [21, 22]. For example, *N*-acetylaspartic acid (NAA) [23–25], creatine [26], and glutamate-glutamine levels [27, 28] have been shown to be reduced in such patients. NAA is a marker of neuronal mitochondria metabolism, as a close association exists between NAA, oxygen, and adenosine triphosphate levels [29, 30]. In addition, Vagnozzi et al. [25] reported that NAA was decreased at 3 and 15 days postinjury and was fully recovered by 30 days. We took this into account on defining the time period of the patient inclusion. We determined that the period within 20 days after injury should be optimal, as NAA levels remained low within this time period after injury.

Similar to MRS, trends of metabolic alterations have been identified using positron emission tomography (PET) studies, including ^18^F-fluorodeoxy glucose (FDG) and ^15^O-labeled O_2_. The results are, however, somewhat inconsistent between studies. Most have reported regional decreases in various areas, but some have reported regional increases or normal metabolism on FDG-PET [31, 32].

Perfusion is another important factor that determines brain temperature. Perfusion is most commonly assessed by single-photon emission computed tomography (SPECT) and PET. The previous works using SPECT reported regional declines in various different areas and also to various degrees [22, 33, 34]. In addition to the inconsistent results from study to study, the lack of quantitative data represents the major limitation of using SPECT in assessing perfusion status.

A more reliable way of performing perfusion assessment will be PET using O^2^, but such studies have not yet been performed for mild trauma cases. Perfusion status in mTBI cases thus remains uncertain. As brain temperature is determined by the balance between metabolism and perfusion, further studies are necessary to determine the true pathophysiology behind this decline in brain temperature.

Body temperature was approximately 0.3 °C higher in the patient group than in the control group in this study. This observation is interesting in light of the relationship between body core and brain temperatures. Brain temperature might be expected to be higher when body core temperature is high, but our observation was the converse. This could further support the assumption of a global decline in brain metabolism.

We also showed that brain temperature may recover after follow-up. However, statistical analysis between the patient and follow-up groups was not significant, possibly owing to the small number of follow-ups. As can be seen in Fig. 1, temperature in the follow-up group showed an increasing trend and appeared to recover to almost the same level as in the control group. Decreased NAA levels after trauma have been reported to recover gradually [25], reflecting improvements in brain metabolism. That report might support our observation of temperature recovery on follow-up. However, the interval until follow-up was variable and the number of patients in the follow-up group was small, so definitive conclusions cannot be drawn from this group.

The novel method shown in this report was proposed by Kozak et al. [12] and is based on the well-known relationship between the diffusivity of free water (unrestricted diffusion) and temperature. Kozak et al. [12] demonstrated this relationship with a water phantom and estimated CSF temperature within the LV using a 3.0-T clinical MR scanner. As DWI is a widely used sequence and is often part of the clinical routine, this method to measure ventricular temperature may be a useful tool in both prospective and retrospective studies. Factors that can positively or negatively impact temperature calculation include the positive effect of the pulsatile movement of the CSF and the negative effect of the coefficient of viscosity. The pulsatile movement of the CSF increases the diffusion coefficient, particularly at specific locations such as the foramen of Monro. We need to examine performing DWI with synchronization to electrocardiograms or CSF flow to account for the effects of the pulsatile movement of the CSF. The reason the coefficient of viscosity is a negative effect is that the diffusion coefficient decreases when the coefficient of viscosity rises. One cause of rises in the coefficient of viscosity for the CSF is an increased protein level. In our study, hemorrhage and meningitis raised the protein level. We cannot completely exclude the possibility of increased protein levels in the CSF in our population, which may have had some effect on measurements. Although we excluded subjects showing intraventricular hemorrhage on T2*-weighted imaging, subjects with subarachnoid hemorrhage on FLAIR at the brain surface were included. We therefore think that hemorrhage represents a potential limitation to this study. Meningitis could also represent a limitation by raising the protein level of the CSF and thus affecting LV or body temperature. However, we did not clinically confirm findings of meningitis in patients with mTBI.

This technique is becoming more widely used. For example, Yamada et al. [13] showed that the ventricular temperature in moyamoya disease patients was 1.1 °C higher than in normal controls. Sakai et al. [14] revealed that LV temperature declines with the normal aging process, while Hasan et al. [35] showed that lateral ventricular ADC was decreased in the elderly. Furthermore, Sai et al. [36] compared temperatures between multiple sclerosis patients and normal controls using this method.

Various limitations need to be considered when interpreting the results of this study. One of these was the low number of patients, particularly of those undergoing follow-up. Further accumulation of follow-up patients is currently underway. Another limitation was that some patients received medications such as antipyretic analgesics (nonsteroidal anti-inflammatory drugs in cases 12, 13, and 19 and steroid in case 17). These medications (particularly antipyretic analgesics) could affect LV temperature or body temperature.

In conclusion, we assessed LV temperature for patients with mTBI using DWI and found a significant decrease in brain core temperature, possibly attributable to a global decline in brain metabolism.
